# Interspecific competition can drive plasmid loss from a focal species in a microbial community

**DOI:** 10.1038/s41396-023-01487-w

**Published:** 2023-08-10

**Authors:** David Sünderhauf, Uli Klümper, William H. Gaze, Edze R. Westra, Stineke van Houte

**Affiliations:** 1 Centre for Ecology and Conservation, University of Exeter, Environment and Sustainability Institute, Penryn, TR10 9FE UK; 2 Department Hydrosciences, Technische Universität Dresden, Institute of Hydrobiology, Dresden, Germany; 3 European Centre for Environment and Human Health, University of Exeter Medical School, Environment and Sustainability Institute, Penryn, TR10 9FE UK

**Keywords:** Microbial communities, Microbial genetics, Bacteria, Soil microbiology, Microbial ecology

## Abstract

Plasmids are key disseminators of antimicrobial resistance genes and virulence factors, and it is therefore critical to predict and reduce plasmid spread within microbial communities. The cost of plasmid carriage is a key metric that can be used to predict plasmids’ ecological fate, and it is unclear whether plasmid costs are affected by growth partners in a microbial community. We carried out competition experiments and tracked plasmid maintenance using a model system consisting of a synthetic and stable five-species community and a broad host-range plasmid, engineered to carry different payloads. We report that both the cost of plasmid carriage and its long-term maintenance in a focal strain depended on the presence of competitors, and that these interactions were species specific. Addition of growth partners increased the cost of a high-payload plasmid to a focal strain, and accordingly, plasmid loss from the focal species occurred over a shorter time frame. We propose that the destabilising effect of interspecific competition on plasmid maintenance may be leveraged in clinical and natural environments to cure plasmids from focal strains.

## Introduction

Plasmids are important mobile genetic elements that facilitate horizontal gene transfer and are crucial components of microbial ecosystems. They shape microbial evolution [1, 2] and are of profound clinical relevance as disseminators of antimicrobial resistance (AMR) genes [3] and virulence factors [4, 5]. Many plasmids, particularly those with a broad host-range, have the potential to transfer between highly diverse bacterial species [6] and mobilise resistance genes from environmental strains into clinically relevant pathogens. Hence, being able to predict and manipulate the spread of plasmids, and of the genes they carry, is critical to limit the spread of AMR.

A key determinant of plasmid spread and maintenance in bacterial populations and communities is the fitness effect that plasmids impose on their bacterial hosts (reviewed in [2]). Costs can arise at different steps of the plasmid lifecycle and can result from, amongst others, the expression of genes carried on the plasmid and their interference with host processes (reviewed in [7]). As a consequence, the cost of plasmid carriage varies not only between plasmids [8] but also between hosts [9]. Moreover, these costs are strongly dependent on the environment; plasmids that are costly in the absence of antibiotics or heavy metals can in turn become highly beneficial in their presence if they encode resistance genes [8].

When plasmids do not encode post-segregational killing systems or active partitioning systems that can prevent plasmid-free segregant formation, theory and data suggest that costly plasmids can be lost readily from bacterial populations or communities due to purifying selection. Plasmid loss can be prevented if conjugation rates—either within or between species—are sufficiently high to support their maintenance [10–12]. For example, bacteria that lose a plasmid when cultured on their own may still associate with this plasmid when co-cultured with another species, due to high rates of interspecific plasmid transfer [10]. Hence, even bacteria that are unable to maintain plasmids in monoculture may experience increased plasmid persistence in a microbial community.

Recent work has shown that the fitness effects of chromosomal mutations in bacteria can depend on the microbial community context [13, 14]. We, therefore, hypothesised that the maintenance of plasmids in focal species might similarly be affected by the microbial community context through amplification or amelioration of the costs of carrying the plasmid. To test this hypothesis, we measured how fitness costs and maintenance of a broad host-range plasmid, pKJK5, in compost isolate *Variovorax* sp. are altered depending on the presence of competitor species in a synthetic five-species community of soil bacterial isolates. These are *Pseudomonas* sp., *Stenotrophomonas* sp., *Achromobacter* sp., and *Ochrobactrum* sp., which form a stable microbial community together with *Variovorax* sp. [15, 16]. We measured cost and maintenance of two variants of pKJK5 with a high and low genetic payload, respectively. After finding that carrying high-payload pKJK5 was only costly to *Variovorax* in the presence of certain growth partners, we measured plasmid maintenance in a series of synthetic communities of varying complexity. The pattern of pKJK5 maintenance was predictable based on the costs of plasmid carriage; *Variovorax* lost pKJK5 more rapidly when embedded in the community. Finally, we generalised community-dependent plasmid loss by measuring pKJK5 maintenance in each constituent of the microbial community and found additional examples where pKJK5 maintenance in constituent species depended on the microbial community context.

## Materials and methods

### Strains and culture conditions

Focal strain *Variovorax* sp. (V) forms a synthetic community with bacterial compost isolates *Pseudomonas* sp. (P), *Stenotrophomonas* sp. (S), *Achromobacter* sp. (A), and *Ochrobactrum* sp. (O). *Variovorax* is a β-proteobacterium, and members of this genus are often found in microbial soil communities. The model species form a stable community in vitro over very long timescales (>1 year) and form visually distinct colonies on King’s B medium (KB) agar, allowing to enumerate species frequencies without the need for selective plating [15]. All communities and monocultures were incubated in 6 mL low-nutrient 1/64 tryptone soy broth (TSB; diluted in water) statically at 28 °C. For analysis, samples were plated onto KB agar plates at appropriate dilutions and incubated at 28 °C for 2–3 days. Community composition was assessed by counting each colony phenotype, ambiguous colony phenotypes were confirmed by 16S rRNA gene colony PCR and Sanger sequencing (using primers 27F 5’-agagtttgatcmtggctcag-3’ and 1492R 5’-accttgttacgactt-3’).

### Engineering of pKJK5::*gfp*^PL^ conjugative plasmid

pKJK5 is a 54-kb IncP1-ε plasmid originally isolated from a manure-associated microbial soil community that carries resistance genes to tetracycline, trimethoprim, aminoglycosides, and sulfonamides within an *intI1* integron cassette [17]. It can readily be transferred into highly diverse members of soil or waste-water treatment plant communities [6, 18].

Using pKJK5 as a template, conjugative plasmid pKJK5::*gfp*^PL^ was constructed by ƛ-red mediated recombineering as described in [19], with the exception that the gene cassette constitutively expressing GFP, *Streptococcus pyogenes* Cas9, and sgRNA was inserted into pKJK5’s *intI1* gene (Supplementary Fig. S1b). The sgRNA is non-functional by encoding a 69-nucleotide guide absent from the study system (gttttctgcctgtcgatccagttttagagctctaaaactggatcgacaggcagaaaacatgtcgatcca). Alongside this, we used pKJK5::*gfp* [6] (Supplementary Fig. S1c).

pKJK5::*gfp*^PL^ and pKJK5::*gfp* were delivered to P, S, A, O, and V using *Escherichia coli* MFD*pir* [20] or *E. coli* K12::mCherry [6] as donors, followed by selecting GFP-expressing (GFP+) transconjugant colonies on KB agar plates containing 12 µg/mL tetracycline only (pKJK5::*gfp*) or tetracycline and 10 µg/mL trimethoprim (pKJK5::*gfp*^PL^).

### *Variovorax* fitness experiment

Two chromosomal tag variants of *Variovorax* were constructed using mini-Tn5-transposon vectors pBAM1-Gm [21] and derivative pBAM1-Cm, which contains chloramphenicol resistance gene *catR* [22]. These suicide transposon vectors were delivered to *Variovorax* using auxotrophic donor strain *E. coli* MFD*pir* following established protocols [22]. Successful insertion of *aacC1* and *catR* genes was confirmed by their resistant phenotype and by PCR.

Five replicate V(Gm^R^) + pKJK5::*gfp*^PL^/pKJK5::*gfp* transconjugant colonies (generated as described above) were suspended in 1/64 TSB + 12 µg/mL tetracycline, and five replicate colonies each of plasmid-free V(Gm^R^), V(Cm^R^), P, S, A, and O were suspended in 1/64 TSB. After the initial 2-day incubation, cultures were washed twice with 0.9% (w/v) NaCl solution to remove all traces of antibiotics before being used to start the experiment. Communities were established by using 20 µL of each *Variovorax* strain (adjusted to OD600 = 0.065) mixed with 50 µL of OD-adjusted P, S, A, or O. OD-adjusted samples were plated onto KB agar for T0 counts. For the pKJK5-bearing treatment, V(Gm^R^) + pKJK5::*gfp*^PL^/pKJK5::*gfp* and V(Cm^R^) competed against each other either alone or together with P, S, A, or O. In the pKJK5-free control, V(Gm^R^) and V(Cm^R^) competed against each other in the same contexts. All competitions were carried out in the absence of antibiotic selection.

The communities were cultured for 3 days, vortexed, and 100 µL were transferred into fresh microcosms and incubated for another 2 days. Then, communities were vortexed and 50 µL of a 10^–5^ dilution of each sample was plated onto KB, KB + 50 µg/mL gentamicin, and KB + 25 µg/mL chloramphenicol plates.

Robustness of chromosomal tags was confirmed by colony PCR of *aacC1* and *catR* of 66 random *Variovorax* colonies across treatments (using primers aacC1-fw 5’-atgttacgcagcagcaacga-3’; aacC1-rv 5’-ttaggtggcggtacttgggt-3’; cm-fw 5’-agacggcatgatgaacctga-3’; cm-rv 5’-cggtgagctggtgatatggg-3’). Colony identities of all species were assessed on each plate. T0 CFU/mL were estimated by multiplying T0 counts with 20/6000 (20 µL of cultures were diluted into 6 mL microcosms). The relative fitness of V(Gm^R^) for each treatment was calculated using the following equation:

$${{{{{{\mathrm{Relative}}}}}}\,{{{{{\mathrm{fitness}}}}}}\,{{W}}\,{{{{{\mathrm{of}}}}}}\,{{{{{\mathrm{V}}}}}}\left( {{{{{\mathrm{{Gm}}}}}}^{{{{{\mathrm{R}}}}}}} \right)} = \frac{{\ln \left( {\frac{{{{{{{{{\mathrm{V}}}}}}}}\left( {{{{{{{{\mathrm{Gm}}}}}}{{{{{\mathrm{R}}}}}}}}} \right){{{{{\mathrm{CFU/mL}}}}}}\,{{{{{\mathrm{at}}}}}}\,T5}}{{{{{{{{{\mathrm{V}}}}}}}}\left( {{{{{{{{\mathrm{Gm}}}}}}{{{{{\mathrm{R}}}}}}}}} \right){{{{{\mathrm{CFU/mL}}}}}}\,{{{{{\mathrm{at}}}}}}\,T0}}} \right)}}{{\ln \left( {\frac{{{{{{{{{\mathrm{V}}}}}}}}\left( {{{{{{{{\mathrm{Cm}}}}}}{{{{{\mathrm{R}}}}}}}}} \right){{{{{\mathrm{CFU/mL}}}}}}\,{{{{{\mathrm{at}}}}}}\,T5}}{{{{{{{{{\mathrm{V}}}}}}}}\left( {{{{{{{{\mathrm{Cm}}}}}}{{{{{\mathrm{R}}}}}}}}} \right){{{{{\mathrm{CFU/mL}}}}}}\,{{{{{\mathrm{at}}}}}}\,T0}}} \right)}}$$

In order to disentangle the effects that growth partners may have on the fitness of *Variovorax* tag variants from the effect of plasmid carriage, relative fitness *W* was normalised by division with relative fitness of pKJK5-free V(Gm^R^) in the same growth context, calculated as above.

One pKJK5-free and one pKJK5::*gfp*^PL^-bearing *Stenotrophomonas* treatment were contaminated with *Achromobacter* colonies and excluded from all visualisations and analyses, resulting in *N* = 4–5.

### Plasmid maintenance experiments

To set up monocultures and communities for the plasmid maintenance experiments, five colonies of each community constituent were individually suspended in 1/64 TSB, supplemented with 12 µg/mL tetracycline where the community constituent carried pKJK5::*gfp*^PL^ or pKJK5::*gfp*. Adding tetracycline at this step ensured pKJK5 maintenance in all strains except *Achromobacter*, which displays resistance to this antibiotic even in the absence of pKJK5.

After 2 days of incubation, 1 mL of these cultures was pelleted and washed twice with 0.9% NaCl to remove all traces of antibiotics. For monocultures, 20 µL of each of the five separately cultured colonies per isolate were transferred into fresh microcosms, giving rise to five biological replicate monocultures per treatment. In addition, 15 community combinations were established by mixing 20 µL of each five replicates of P, S, A, and/or O and V, giving rise to five replicate communities per treatment (Supplementary Fig. S2b). All experiments were carried out in the absence of antibiotic selection.

Monocultures and communities were cultured for 3 days, vortexed, and 100 µL of each culture was transferred into a fresh microcosm and incubated for another 2 days. Communities were then further passaged into fresh microcosms the same way every 2 days until 17 total days of co-culture (community maintenance experiment only). To assess community composition, 50 µL of a 10^–5^ dilution of samples were plated onto KB agar plates at T0, T5, and T17 (Supplementary Fig. S2c). Plasmid carriage was assessed by screening for GFP expression using a fluorescence lamp (NightSea lamp with RB bandpass filter).

### Statistical analyses

Data processing, data visualisation, and statistical analyses were carried out using R version 4.0.5 and RStudio version 1.4.1103 with the following packages: dplyr, tidyr, readr, ggplot2, ggpubr, MuMIn, mgcv, gamm4, ggeffects, GGally, and MASS.

For all analyses, other model types, link functions, and the inclusion of additional variables were tested. The final models were found to be the most robust. Model assumptions were checked and found to be upheld. For comparison of specific treatment categories, Tukey’s post-hoc test of honest significance differences was carried out.

#### *Variovorax* fitness

We carried out one-sample *t*-tests for each normalised *W* to test for a significant difference from one, which indicates no fitness change relative to a pKJK5-free growth context. *T*-tests were carried out on log-transformed data and therefore tested for differences to zero. To account for multiple testing within the same dataset, after a Bonferroni adjustment, the significance threshold was considered as *p* = 0.01.

*p* values for fitness cost of pKJK5::*gfp*^PL^ were as follows: in monoculture *p* = 0.1367; with *Pseudomonas p* = 0.02282; with *Stenotrophomonas p* = 0.004755; with *Achromobacter p* = 0.7507; with *Ochrobactrum p* = 0.07439.

*p* values for fitness benefit of pKJK5::*gfp* were as follows: in monoculture *p* = 0.02136; with *Pseudomonas p* = 0.002179; with *Stenotrophomonas p* = 0.9101; with *Achromobacter p* = 0.2844; with *Ochrobactrum p* = 0.6933.

#### Growth partner experiment

We explored pKJK5::*gfp*^PL^ data by fitting five individual LMs, each describing *Variovorax* plasmid maintenance as a function of species prevalence. For each model, datapoints with a prevalence of zero were dropped. Model statistics for each model were as follows: *Pseudomonas* proportion: *F* = 0.02; df = 1 and 38; *p* = 0.90; *R*^2^ = 0.00038; *Stenotrophomonas* proportion: *F* = 4.4; df = 1 and 38; *p* = 0.042; *R*^2^ = 0.10; *Achromobacter* proportion: *F* = 18.1; df = 1 and 38; *p* = 0.00013; *R*^2^ = 0.32; *Ochrobactrum* proportion: *F* = 2.9; df = 1 and 38; *p* = 0.095; *R*^2^ = 0.072; *Variovorax* proportion: *F* = 5.2; df = 1 and 78; *p* = 0.025; *R*^2^ = 0.063.

Fitting an LM between *Variovorax* proportion and plasmid maintenance without the monoculture data showed no association (*F* = 0.0006; df = 1 and 73; *p* = 0.98; *R*^2^ = 8.25 × 10^–6^). Effect sizes were obtained from the models’ coefficient estimates.

We carried out co-variation analyses using package GGally and fitted a single GLM to the full dataset to avoid multiple testing. As only four (*N* – 1) variables of community member proportion could be fitted to the data simultaneously, we initially constructed five binomial GLMs describing pKJK5-bearing *Variovorax* fraction as a function of treatment and four community constituent proportions, leaving out each in turn. In these models, *Ochrobactrum* proportion never reached statistical significance. Therefore, we constructed a GLM lacking this variable as a starting point and successively reduced the model to only include significant variables. See Supplementary Table S3 for a summary of which additional model variables were dropped due to insignificance.

The final model was a binomial GLM with logit link function. Plasmid-bearing *Variovorax* fraction was modelled as a function of Treatment (community), *Stenotrophomonas* proportion, and *Achromobacter* proportion. Total *Variovorax* colony counts for each sample were taken into account as weights. Model statistics are as follows: *F* = 0.5, df = 17 and 62, pseudo *R*^2^ = 0.85.

The model estimates are shown in Supplementary Table S4.

#### Plasmid maintenance-fitness correlation

The dataset combines relative fitness data with plasmid maintenance data. To synthesise matching treatments, we built a binomial generalised linear mixed-effects model with probit link function using replicates from each experiment as random effects. This was done to statistically investigate all datapoints, rather than just arithmetic means without aligning these metrics for each replicate across the two experiments.

pKJK5-bearing *Variovorax* fraction was modelled as a function of log-transformed normalised relative fitness *W*, with random intercept effects of fitness experiment replicate and maintenance experiment replicate. Overall *Variovorax* colony counts were taken into account as weights. Model statistics are as follows: *F* = 40.9; 50 observations with 2 × 10 random-effect groups, conditional *R*^2^ = 0.50, marginal *R*^2^ = 0.21. Relative fitness *W* is a significant constituent of the model (*p* = 7.13 × 10^–11^).

#### Community maintenance experiment

A single extremely influential datapoint was removed for statistical analyses (P+pKJK5::*gfp* community treatment at T5; the only replicate where *Pseudomonas* was detected with one GFP-colony). Some monoculture samples were contaminated with colonies of other species. These replicates and subsequent timepoints were entirely removed for all data visualisation and analyses, resulting in *N* = 3–5 for all treatments, except A carrying pKJK5:*gfp* which has two replicates at T17. The affected samples are as follows: for pKJK5::*gfp*: P replicate 1 from T0; S replicate 2 from T17; A replicate 3 from T0, replicate 2 from T5, and replicate 4 from T17; V replicate 4 from T5. For pKJK5::*gfp*^PL^: P replicate 1 from T17; O replicate 1 from T17.

We constructed a binomial GLM with logit link function for each timepoint: pKJK5-bearing colony fraction was modelled as a function of species, culture conditions (monoculture/community), plasmid type (pKJK5::*gfp*/pKJK5::*gfp*^PL^), and their interactions. Total focal species colony counts were taken into account for each sample as weights. Both models’ statistics are as follows: T5: *F* = 1.5, df = 18 and 71, pseudo *R*^2^ = 0.99. T17: *F* = 0.6, df = 19 and 70, pseudo *R*^2^ = 0.98.

## Results

### pKJK5 was costly to *Variovorax* in the presence of growth partners

We hypothesised that the cost of carrying the broad host-range, IncP1ε plasmid pKJK5 in *Variovorax* sp. depends on the microbial community context. To test this, we measured the fitness costs of carrying the plasmid by competing pKJK5-bearing *Variovorax* that was chromosomally tagged with gentamicin resistance gene *aacC1* (V(Gm^R^)) with pKJK5-free *Variovorax* that was chromosomally tagged with chloramphenicol resistance gene *catR* (V(Cm^R^)). As a control, we also competed pKJK5-free V(Gm^R^) with pKJK5-free V(Cm^R^). Clones were competed either in isolation or in the presence of each of the four different growth partners (*Pseudomonas* sp*., Stenotrophomonas* sp*., Achromobacter* sp., or *Ochrobactrum* sp.) in five biological replicates. Relative fitness *W* of V(Gm^R^) in pKJK5-bearing conditions was normalised by *W* in matching pKJK5-free conditions to account for any differential effects growth partner presence may have on the two *Variovorax* tag variants. To enable visualisation of plasmid transfer, we measured cost of plasmid carriage of two different green fluorescent protein (GFP)-tagged versions of this plasmid. pKJK5::*gfp*^PL^ also encodes constitutively expressed *cas9* and a non-targeting *sgRNA* (Supplementary Fig. S1b) to facilitate microbial community engineering in the future [23]. In this work, these genes act as payload (PL) genes. Alongside this, we used pKJK5::*gfp*, a plasmid lacking these additional payload genes (Supplementary Fig. S1c).

**Fig. 1:**
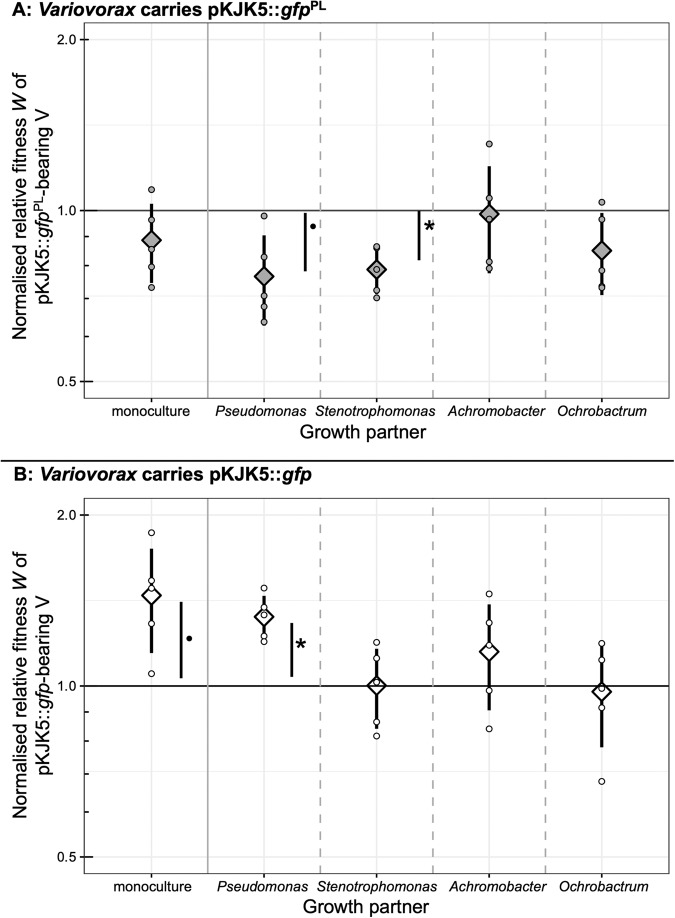
Plasmid cost to *Variovorax* is dependent on growth context. Mean ± standard deviation (including individual datapoints) of fitness *W* of pKJK5-bearing *Variovorax* relative to a pKJK5-free *Variovorax* tag variant, adjusted for relative fitness of these strains in an entirely pKJK5-free matching treatment. The black line (1) indicates no change of fitness relative to pKJK5-free conditions. **A** *Stenotrophomonas* presence as a growth partner leads to a significant cost of pKJK5::*gfp*^PL^ carriage to *Variovorax; p* = 0.0048. **B** *Pseudomonas* presence as a growth partner leads to a significant benefit of pKJK5::*gfp* carriage to *Variovorax*; *p* = 0.0022. Significance tested for with one-sample *t*-tests on log-transformed data. •*p* < 0.05, non-significant; *p < 0.01, significant after Bonferroni adjustment for multiple testing. All other values are *p* > 0.05.

Relative fitness of *Variovorax* carrying pKJK5::*gfp* was consistently higher than that of *Variovorax* carrying pKJK5::*gfp*^PL^ in all growth contexts, indicating a lower overall cost of the plasmid lacking payload genes. These competition experiments further revealed that pKJK5::*gfp*^PL^ carriage was associated with a fitness cost to *Variovorax* in the presence of *Stenotrophomonas* (Fig. 1A; normalised *W* < 1 means the plasmid is costly in this growth context; *p* = 0.0048; significant after one-sample *t*-test and Bonferroni adjustment). pKJK5::*gfp*^PL^ did not impose a significant fitness cost on *Variovorax* in other growth contexts in this experiment. This demonstrates that after 5 days of growth, carrying pKJK5::*gfp*^PL^ was only costly to *Variovorax* in the presence of *Stenotrophomonas*.

Unlike the plasmid carrying payload genes, pKJK5::*gfp* was not costly to *Variovorax* in any growth context (Fig. 1B). Instead, pKJK5::*gfp* provided a fitness benefit to *Variovorax* when growing with *Pseudomonas* (*p* = 0.0022), a pattern not observed for the costlier pKJK5::*gfp*^PL^. Fitness during all other growth contexts did not depend on pKJK5::*gfp* carriage.

Analysis of GFP expression of *Variovorax* colonies on various selective plates revealed that the frequencies of conjugation of both plasmids were very low and stochastic under our experimental conditions, with only eight out of 983 or five out of 509 colonies of the initially pKJK5-free *Variovorax* strain becoming GFP positive (~0.81/0.98% transconjugants/recipients for pKJK5::*gfp*^PL^ and pKJK5::*gfp* respectively; Supplementary Table S1). With two exceptions where three and two transconjugant colonies respectively were found in the same replicate, all *Variovorax* transconjugants found were single colonies within replicates. Other species never formed transconjugants. Together, this confirms that conjugation is not relevant to this study system and that, instead, it allows us to investigate the impact of fitness on plasmid maintenance in isolation.

### Plasmid costs were linked to maintenance

Based on the increased cost of pKJK5::*gfp*^PL^ to *Variovorax* in the presence of *Stenotrophomonas*, we hypothesised that co-culture with growth partners would also lead to decreased plasmid maintenance. To test this, we generated 16 different synthetic microbial communities with five replicates each, composed of all possible combinations of one, two, three, four, and five species, consisting of *Variovorax* carrying pKJK5::*gfp*^PL^ and pKJK5-free *Pseudomonas*, *Stenotrophomonas*, *Achromobacter*, and/or *Ochrobactrum*. To give us a full picture of plasmid maintenance and its associated fitness, we carried out the same experiment with pKJK5::*gfp*. We measured plasmid maintenance within the focal strain *Variovorax* after 5 days of co-culture (Supplementary Fig. S2).

While different communities in which *Variovorax* carried pKJK5::*gfp*^PL^ showed variation in *Variovorax* plasmid maintenance (Fig. 2A), communities in which *Variovorax* carried the plasmid lacking payload genes did not (Fig. 2B). Therefore, we further investigated maintenance of only pKJK5::*gfp*^PL^ after this experiment.

**Figure:**
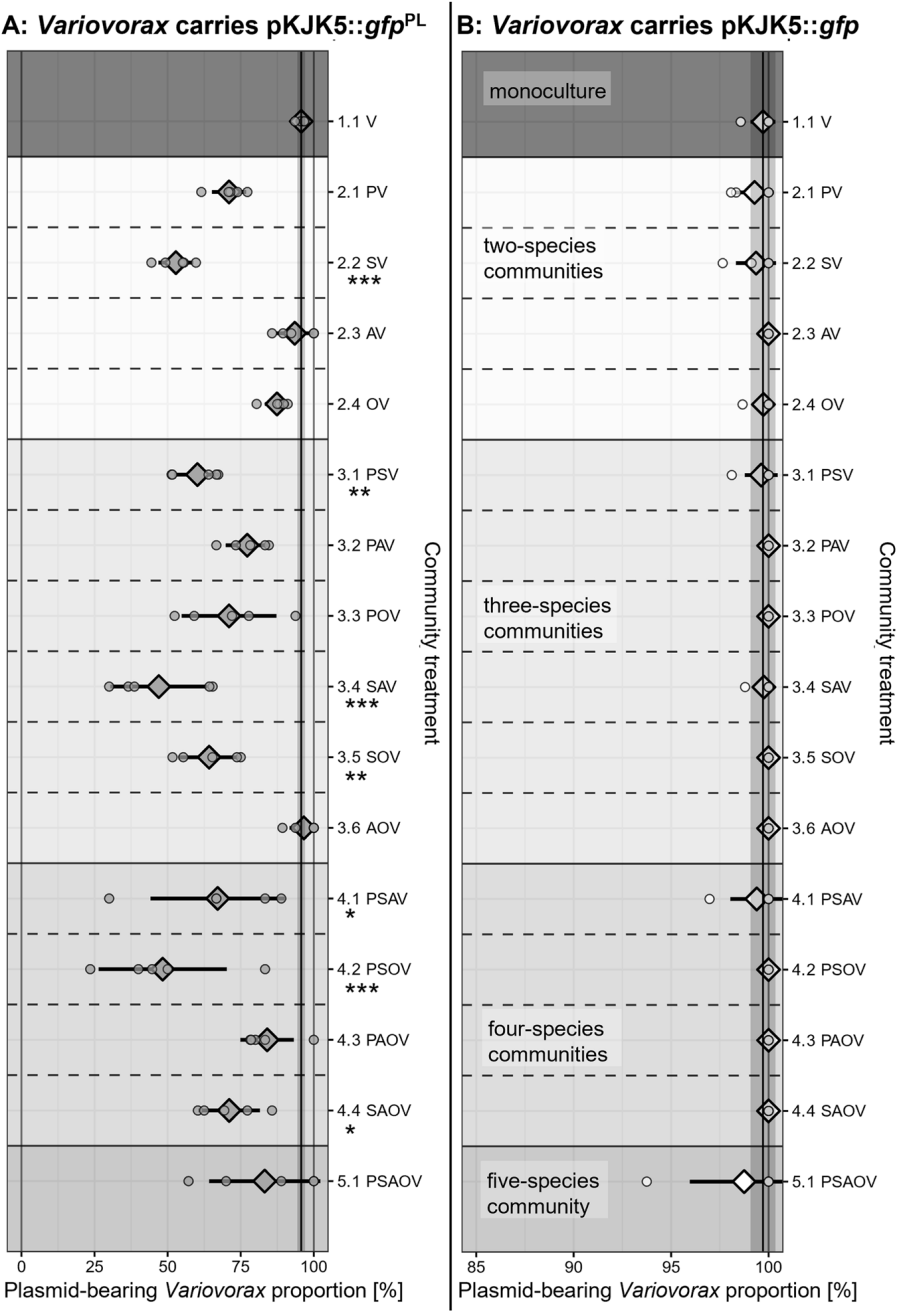
Fig. 2 Different combinations of growth partners elicit different *Variovorax* plasmid loss effects. Mean ± standard deviation (including individual datapoints) of GFP+ *Variovorax* (V) fraction as a proxy of plasmid-bearing *Variovorax* in the presence of various growth partners (*Pseudomonas* (P), *Stenotrophomonas* (S), *Achromobacter* (A), *Ochrobactrum* (O)) after 5 days of co-culture. All possible community combinations were assembled for *Variovorax* carrying either pKJK5::*gfp*^PL^ (**A**) or pKJK5::*gfp* (**B**). Note that the axis for (**B**) starts at 85%. For comparison, the vertical black line and shaded areas indicate the mean ± standard deviation of the monoculture treatment. Stars indicate treatments with significantly lower GFP+ fraction than in monoculture. See Supplementary Table S1 for values of these summary statistics for pKJK5::*gfp*^PL^. *p < 0.05, **p < 0.01, ***p < 0.001 as calculated by Tukey’s HSD after fitting a binomial GLM; *F* = 0.5; df = 17 and 62; pseudo *R*^2^ = 0.85; *N* = 5.

In monoculture, nearly all *Variovorax* clones in the population retained pKJK5::*gfp*^PL^ (Fig. 2A). In contrast, most communities showed decreased plasmid maintenance in *Variovorax*. To further delve into this effect and to determine statistical significance, we wanted to explore whether the prevalence of certain growth partners influenced pKJK5::*gfp*^PL^ maintenance in *Variovorax*. We visualised this by fitting separate linear models (LMs) to each subset of treatments, excluding those which lacked the species being investigated (Table 1 and Supplementary Fig. S3).

**Table 1:** Effects of abundance of each community member in isolation on *Variovorax* pKJK5::*gfp*^PL^ maintenance.

Community member	Significance	Explained variation	Effect on *Variovorax* pKJK5::*gfp*^PL^ maintenance
*Pseudomonas sp*.	*p* = 0.90	*R*^*2*^ = 0.00038	0.019%
*Stenotrophomonas sp*.	*p* = 0.042*	*R*^*2*^ = 0.10	−0.30%
*Achromobacter sp*.	*p* = 0.00013***	*R*^*2*^ = 0.30	0.49%
*Ochrobactrum sp*.	*p* = 0.095	*R*^*2*^ = 0.072	0.36%
*Variovorax sp*.	*p* = 0.025*	*R*^*2*^ = 0.063	0.21%
*Variovorax sp*. (monoculture treatment excluded)	*p* = 0.98	*R*^*2*^ = 8.3 × 10^–6^	−0.0041%

Significance (*p* value; *p < 0.05, ***p < 0.001), explained variation (*R*^*2*^), and effect on *Variovorax* pKJK5::*gfp*^PL^ maintenance (derived from model coefficients, % change in plasmid maintenance for a 1% rise in community member abundance) of individual linear models fitted to data in Fig. 2A. Communities where community members were entirely absent were excluded from each model. Effects of *Variovorax* abundance were likely driven by the monoculture treatment only; therefore, a model with this treatment excluded is presented as well. These models are visualised in Supplementary Fig. S3. Only *Stenotrophomonas* and *Achromobacter* proportion constitute significant terms of the statistical model fitted to the full dataset; see Supplementary Table S3.

These LMs showed that (after removal of the highly influential monoculture treatment) *Stenotrophomonas* and *Achromobacter* abundance correlated with *Variovorax* plasmid maintenance. There was a clear negative association between the proportion of *Stenotrophomonas* and the plasmid-bearing *Variovorax* fraction (−0.3%). Interestingly, *Achromobacter* had the opposite effect: its presence was associated with higher plasmid maintenance (+0.49%). To avoid multiple testing and account for correlations between various community members (such as a negative correlation between *Stenotrophomonas* and *Achromobacter* proportions; Supplementary Fig. S3), we iteratively tested which community member presence influences *Variovorax* plasmid maintenance in a single statistical model (Supplementary Table S3).

The full statistical model revealed that only *Stenotrophomonas* (*p* = 0.0032; binomial GLM, *F* = 0.5, df = 17 and 62, pseudo *R*^*2*^ = 0.85) and *Achromobacter* (*p* = 0.0016) proportion had statistically significant effects on *Variovorax* plasmid maintenance, while the proportion of all other community members did not significantly influence the pKJK5-bearing *Variovorax* fraction. Compared with the monoculture treatment, *Variovorax* plasmid maintenance was significantly decreased in seven of the 16 synthetic communities (*p* < 0.05; see Supplementary Table S2 for a full breakdown) Fitting with the above observations, these corresponded to all those communities that contained *Stenotrophomonas*, with the sole exception of the full five-species community where the reduction in *Variovorax* plasmid maintenance was not significant (*p* = 0.65).

**Fig. 3:**
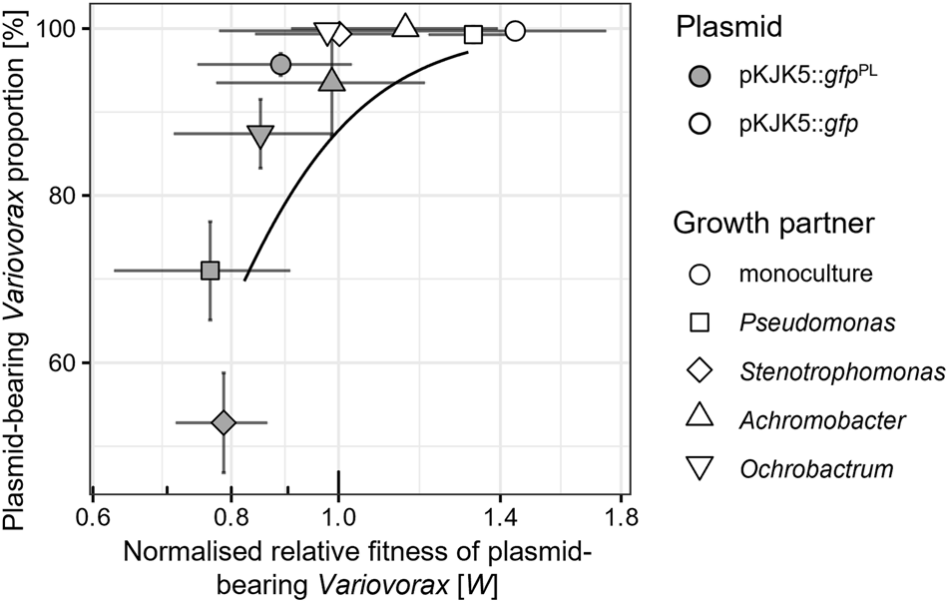
Plasmid cost to *Variovorax* is a significant predictor of plasmid maintenance across growth contexts. Mean ± standard deviation of pKJK5 cost to *Variovorax* (logarithm of relative fitness *W*, normalised by plasmid-free *W* in the same growth context) as in Fig. 1 and pKJK5-bearing *Variovorax* fraction in corresponding treatments as in Fig. 2. The black line indicates the fitted mixed-effects binomial model. Total number of colonies counted was taken into account as weights in modelling. *F* = 40.9, 50 observations with 2 × 10 random-effect groups, marginal *R*^2^ = 0.21, conditional *R*^2^ = 0.50. Fitness *W* is a significant determinant of plasmid-bearing *Variovorax* fraction, *p* < 0.001.

To formally address the hypothesis that the differences in plasmid maintenance across the synthetic communities were associated with differences in the cost of plasmid carriage to *Variovorax*, we performed a correlational analysis between cost of carriage of either pKJK5::*gfp*^PL^ or pKJK5:*gfp* to *Variovorax* (Fig. 1) and maintenance of these plasmids (Fig. 2). Treatments in which the relative fitness of pKJK5-bearing *Variovorax* was higher also displayed higher levels of *Variovorax* plasmid maintenance (Fig. 3; *p* < 0.001, mixed-effects binomial GLM, *F* = 40.9, 50 observations, 2 × 10 random-effect groups, conditional *R*^2^ = 0.50, marginal *R*^2^ = 0.21).

While this analysis does not allow us to predict plasmid maintenance from plasmid cost, or indeed explain most of the observed variation of plasmid maintenance, it does reveal a clear association between these two metrics. All pKJK5::*gfp* treatments, the pKJK5::*gfp*^PL^ monoculture treatment and community treatments consisting of either *Achromobacter* or *Ochrobactrum* as a growth partner clustered in the top-right, representing treatments where plasmid cost was low and plasmid maintenance high. In contrast, pKJK5::*gfp*^PL^ community treatments that contained either *Pseudomonas* or *Stenotrophomonas* as a growth partner were associated with low plasmid maintenance and higher costs of plasmid carriage to *Variovorax*.

### pKJK5 maintenance was community-dependent in multiple species

After linking plasmid cost to plasmid maintenance within *Variovorax*, and showing how these were altered by embedding the host in a community context, we sought to explore whether the observed effects were unique to *Variovorax* and pKJK5::*gfp*^PL^ or if they could be observed in other host-plasmid pairs.

**Fig. 4:**
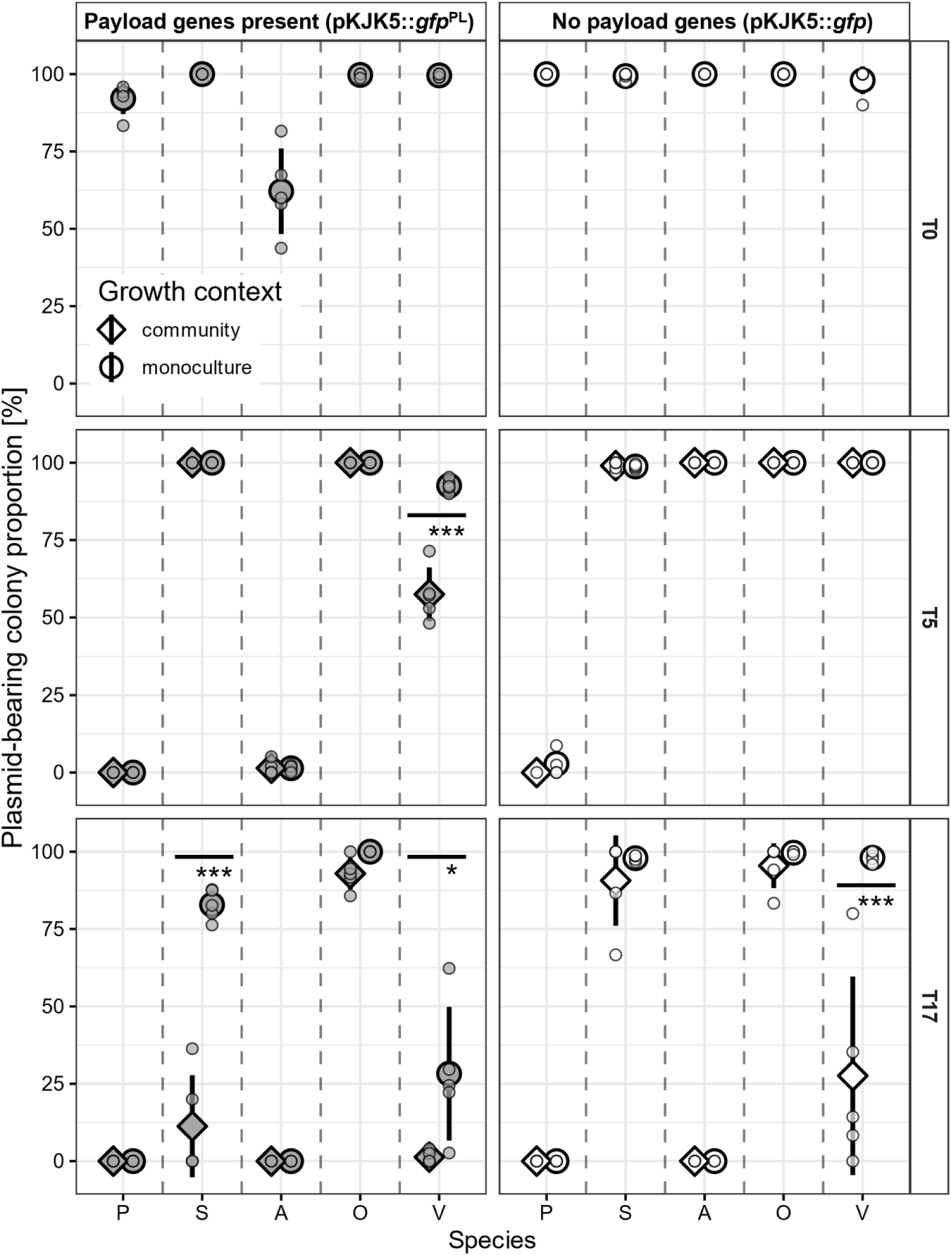
*Variovorax* and *Stenotrophomonas* pKJK5 maintenance is dependent on community context. Mean ± standard deviation (including individual datapoints) of GFP+ fraction of colonies as a proxy of pKJK5::*gfp*^PL^ (left) or pKJK5::*gfp* (right) maintenance. Data are split into species *Pseudomonas* (P), *Stenotrophomonas* (S)*, Achromobacter* (A), *Ochrobactrum* (O), and *Variovorax* (V) after 5 and 17 days of growth in monoculture or in a community context. T0 indicates pKJK5-maintaining proportion of strains used to start the experiment. **p < 0.01, ***p < 0.001 as calculated by Tukey’s HSD after fitting binomial GLMs; T5: *F* = 1.5; df = 18 and 71; pseudo *R*^2^ = 0.99; T17: *F* = 0.6; df = 19 and 70; pseudo *R*^2^ = 0.98; *N* = 4–5. No other treatment combinations are significantly different from each other.

We carried out plasmid maintenance experiments in which all species carried the plasmid (*Pseudomonas, Stenotrophomonas, Achromobacter, Ochrobactrum, Variovorax*), either in the presence or absence of additional payload genes (pKJK5::*gfp*^PL^ and pKJK5::*gfp*, respectively). The frequencies of pKJK5::*gfp*^PL^ and of pKJK5::*gfp* carriage were measured over 17 days in each host cultured in isolation or as part of a stable microbial community consisting of all five species bearing the plasmid. For both pKJK5 variants, maintenance of the plasmid strongly depended on host identity; *Ochrobactrum* retained pKJK5::*gfp* and pKJK5::*gfp*^PL^ to high levels, while *Pseudomonas* and *Achromobacter* lost these plasmids during the course of the experiment (Fig. 4). These differences in plasmid maintenance were independent of the community context in which the hosts were cultured.

Unlike *Pseudomonas* or *Achromobacter* plasmid maintenance, *Variovorax* plasmid maintenance was dependent on the community context it is cultured in. In monoculture, 93 ± 2% of colonies retained pKJK5::*gfp*^PL^ after 5 days. In contrast, significantly fewer (57 ± 9%) *Variovorax* clones within the community retained pKJK5::*gfp*^PL^ (*p* < 0.001; binomial GLM; *F* = 1.5; df = 13 and 73; pseudo *R*^2^ = 0.99). This effect remained evident after 17 days of culture (28 ± 22% *Variovorax* plasmid maintenance in monoculture, 1.3 ± 1.9% maintenance in community context). Despite its fitness benefit to *Variovorax* in some two-species communities, similar dynamics could be observed for *Variovorax* carrying pKJK5::*gfp* after 17 days, where 98 ± 2% of colonies retained pKJK5::*gfp* in monoculture compared to 28 ± 32% of colonies in a community context when all growth partners were present. This could indicate that even where a fitness cost of pKJK5::*gfp* carriage could not be observed in simple communities, the plasmid becomes costlier to *Variovorax* in a community consisting of five species. In addition, significant pKJK5::*gfp*^PL^ plasmid loss from *Variovorax* had not been observed during the previous experiment when growth partners did not carry the plasmid (Fig. 2A; treatment 5.1). Therefore, these results also highlight how context-dependent plasmid maintenance was: whether or not growth partners also carried a plasmid had an impact on community structure (Supplementary Fig. S6), which in turn affected pKJK5::*gfp*^PL^ maintenance outcome in *Variovorax*.

Plasmid maintenance in *Stenotrophomonas* was high for both pKJK5 variants after 5 days, independent of community context. However, after 17 days, *Stenotrophomonas* pKJK5::*gfp*^PL^ maintenance remained significantly higher in monoculture (83 ± 5%) than in a community context (11 ± 16%; *p* < 0.001; binomial GLM; F = 0.6; df = 19 and 74; pseudo *R*^2^ = 0.98). While no statistical difference of *Stenotrophomonas* pKJK5::*gfp* maintenance was observed, average maintenance of pKJK5::*gfp* was slightly lower in the community context after 17 days of growth (91 ± 15% maintenance in the community vs. 98 ± 1% maintenance in monoculture).

Collectively, we observed qualitatively similar plasmid maintenance dynamics for pKJK5::*gfp*^PL^ and pKJK5::*gfp* in all host species, exemplified by similar dynamics in pKJK5::*gfp* maintenance at T17 and pKJK5::*gfp*^PL^ maintenance at T5. pKJK5::*gfp*^PL^’s payload genes *cas9* and non-targeting *sgRNA* (Supplementary Fig. S1b) are known to carry a constitutive cost to the bacterial host [24–26], and we confirmed this by assessing the relative fitness of *Variovorax* carrying each plasmid (Fig. 1). The smaller payload of pKJK5::*gfp* slowed plasmid loss dynamics in comparison to costlier pKJK5::*gfp*^PL^. Furthermore, the presence of a microbial community accelerated loss of plasmid pKJK5::*gfp*^PL^ in multiple species of this synthetic community.

## Discussion

This work aimed to understand how costs and maintenance of a conjugative plasmid can be determined by the microbial community context of its host by using a model community composed of five different species of soil bacteria. These species form a locally maladapted community in which the most common form of community interactions is resource competition. No bacterial warfare in the form of direct growth inhibition or killing of growth partners takes place in this system, and all community members can grow to an equilibrium concentration when invading from rare [15, 16]. Consistent with this, species abundance data during our experiments indicate that community dynamics are driven by competitive interactions, as the density of each species remained lower in the community than in monoculture (Supplementary Fig. S7). Under culture conditions which favour long-term species coexistence, little to no conjugation took place (Supplementary Table S1). Therefore, this model system allowed us to disentangle plasmid cost from conjugation impacts on plasmid maintenance.

Using this model community, we found that the fitness cost of plasmid carriage can change as a result of interspecific competition. Embedding focal strain *Variovorax* sp. in a community led to increased costs of pKJK5::*gfp*^PL^ plasmid carriage and more rapid loss of this plasmid. This relationship between plasmid maintenance and plasmid cost is further exemplified by more rapid plasmid loss when a plasmid with payload genes and higher intrinsic cost was used (Fig. 4; pKJK5::*gfp*^PL^ was lost more rapidly than pKJK5::*gfp*). An absence of payload genes on pKJK5::*gfp* switched pKJK5::*gfp*’s fitness effect on *Variovorax* in some growth contexts: growth together with *Pseudomonas* led to the benefit of plasmid carriage. Such a benefit may be explained by the weaponization of costly plasmid carriage by *Varivoroax* (where pKJK5::*gfp* would provide a greater fitness detriment to a *Pseudomonas* transconjugant than to its original host [27]), or by wider-reaching impacts of plasmid carriage on *Variovorax* host physiology (exemplified by markedly different colony morphology of pKJK5-bearing *Variovorax* from pKJK5-free colonies).

The mechanism of plasmid loss in our system is unknown, but both higher intrinsic plasmid loss rates and higher relative growth of plasmid-free segregants would lead to the same observed outcome of population-level plasmid loss. A higher relative growth of plasmid-free segregants can directly be driven by the increased cost of plasmid carriage. This increased cost may be a result of resource limitation during growth with competitors.

Generally, plasmid maintenance is dependent not only on fitness costs and benefits to its host, but also the plasmid transfer and loss rate [28, 29]. Of these, plasmid transfer can also depend on the community context: A mercury resistance plasmid was rapidly lost from its host species in monoculture, but with growth partners, the plasmid was maintained in the focal strain due to reinfection by conjugation [10, 30]. This phenomenon of plasmid persistence through conjugation has been observed for multiple types of plasmids, and in communities consisting of several *E. coli* strains and several plasmids [29]. Ultimately, in these experiments, embedding a plasmid host in a community context led to increased plasmid maintenance as a result of increased conjugation. The fitness cost of the plasmids was not a good predictor of plasmid maintenance [10]. In contrast, in our experiments, the relative fitness during plasmid carriage was a significant determinant of plasmid maintenance outcome; therefore, we propose that a trade-off between plasmid cost and plasmid conjugative transfer may determine plasmid maintenance outcome. Which of these variables has higher importance depends on the study system: we speculate that plasmid maintenance in a focal species is increased in communities with high plasmid conjugation frequencies by providing a reservoir of plasmid-containing cells that re-infect other bacteria in the community. In turn, plasmid maintenance is decreased in communities with low conjugation frequencies, where interspecific competition can increase the cost of plasmid carriage.

Further to a trade-off between plasmid transfer and cost in its maintenance, recent work indicates that community context can also limit conjugation to focal species due to the dilution effect [31]. Both plasmid cost and transfer are also subject to evolution, which can be specific to each plasmid-host pair [32]. While we did not assess this in our study system, constrained evolution of *Variovorax* whilst competing with growth partners could explain different outcomes of plasmid cost and maintenance in monoculture and community contexts. In addition, a study found that plasmid fitness costs vary for different plasmid hosts, and that this fitness cost variability can explain plasmid persistence in diverse communities, due to an increased likelihood of the community containing a permissive host which readily maintains the plasmid [9]. A variability of fitness cost is supported in our own results of plasmid maintenance (Fig. 4), and the study’s opposing conclusion highlights how the same plasmid maintenance dynamics lead to different observational outcomes when shifting focus from a community level to a focal species level. In our work, plasmid maintenance in *Variovorax* decreased when increasing community diversity from one to five species, but overall, the plasmid was maintained on a community level even at high diversity due to *Ochrobactrum’s* permissive nature.

Overall, this study and previous work emphasise the importance of variable plasmid costs and plasmid reinfection rates when considering plasmid maintenance in a focal host species within a community context.

While we did not generalise this effect beyond our study system, it is highly unlikely that only *Variovorax* and *Stenotrophomonas* maintain plasmids in a community-dependent manner. Provided we have sufficient knowledge about community interactions and plasmid behaviour to predict the outcome of embedding a focal strain in a community context, community-dependent plasmid loss could have wider implications: in healthcare, bacteria might lose a plasmid-containing virulence factors or antibiotic resistance genes in a new community context. For instance, a plasmid present in environmental microbiomes might become lost once the plasmid host becomes embedded in a human microbiome, as observed when a virulence plasmid became lost from *E. coli* in the human gut during the course of an infection [33], although the mechanisms behind plasmid loss were not investigated in this study. Conversely, resistance plasmids which are maintained in a community context may be lost in diagnostic monocultures. Community-level plasmid loss would especially need to be investigated if previously characterised highly permissive plasmid hosts, which are key to plasmid maintenance on a community level [18], are shown to experience community-dependent impacts on their plasmids’ fitness cost (analogous to highly successful plasmid-host pairs presenting a low fitness cost and high conjugation rates [34]).

Further to explaining natural phenomena of plasmid maintenance and loss, this work could have implications on the spread of synthetic plasmids bearing payload genes through microbial communities (e.g., CRISPR-Cas antimicrobials [23]). Where payload genes increase the plasmid’s fitness cost, this may impact its maintenance in a microbial community even when the plasmid can be stably maintained in single bacterial strains. Finally, this work opens exciting research avenues for the manipulation of plasmid content of focal species. For example, removal of virulence and resistance plasmids from pathogens may be achieved through the addition of certain plasmid loss-inducing growth partners. This prediction needs to be tested thoroughly using additional plasmid-host pairs.

## Supplementary information

Supplementary Figures and Tables

Supplementary Data

## Data Availability

All data generated during this study are included in the supplementary information files.
