# Supplementary material for: Interspecific competition can drive plasmid loss from a focal species in a microbial community
Source: ISME J. 2023 Aug 10;17(10):1765–73. doi: 10.1038/s41396-023-01487-w (PMC10504238; doi:10.1038/s41396-023-01487-w)
Supplement: Supplementary file 1 — Supplementary Figures and Tables [file 41396_2023_1487_MOESM1_ESM.docx]

**Interspecific competition can drive plasmid loss from a focal species in a microbial community.**

David Sünderhauf^1^*, Uli Klümper^2^, William H Gaze^3^, Edze R Westra^1^, Stineke van Houte^1^*.

1. Centre for Ecology and Conservation, University of Exeter, Environment and Sustainability Institute, Penryn, UK, TR10 9FE

2. Department Hydrosciences, Technische Universität Dresden, Institute of Hydrobiology, Dresden, Germany

3. European Centre for Environment and Human Health, University of Exeter Medical School, Environment and Sustainability Institute, Penryn, UK, TR10 9FE

*corresponding authors; [david@sunderhauf.net](mailto:david@sunderhauf.net); [c.van-houte@exeter.ac.uk](mailto:c.van-houte@exeter.ac.uk)

**Supporting Information**

Supplementary Data: Raw colony counts and relative fitness data from the pKJK5 fitness experiment (Figure 1), and raw colony counts from the community maintenance experiments (Figures 2 and 4). These are available in separate datasheets as a supplementary excel file.

Table S1: Conjugation efficiencies to *Variovorax* during the fitness experiment. Conjugation efficiency, defined as the final proportion of transconjugants within the recipient population, after 5 days of co-culture is given for each sample. Other species did not form transconjugants.

| **Growth partner** | **Replicate** | **Donors** | ***Variovorax* recipients** | **Transconjugants** | **Conjugation efficiency** |
| --- | --- | --- | --- | --- | --- |
| pKJK5::*gfp*^PL^ transfer | | | | | |
| monoculture | A | 38 | 65 | 0 | 0 |
| monoculture | B | 28 | 81 | 0 | 0 |
| monoculture | C | 20 | 43 | 0 | 0 |
| monoculture | D | 54 | 48 | 0 | 0 |
| monoculture | E | 21 | 58 | 0 | 0 |
| *Pseudomonas* | A | 19 | 24 | 0 | 0 |
| *Pseudomonas* | B | 20 | 48 | 0 | 0 |
| *Pseudomonas* | C | 12 | 30 | 1 | 3.33% |
| *Pseudomonas* | D | 15 | 36 | 0 | 0 |
| *Pseudomonas* | E | 20 | 31 | 2 | 6.45% |
| *Stenotrophomonas* | A | 9 | 40 | 1 | 2.50% |
| *Stenotrophomonas* | B | 45 | 68 | 1 | 1.47% |
| *Stenotrophomonas* | C | 19 | 38 | 0 | 0 |
| *Stenotrophomonas* | D | 20 | 34 | 0 | 0 |
| *Stenotrophomonas* | E | 20 | 53 | 0 | 0 |
| *Achromobacter* | A | 7 | 23 | 0 | 0 |
| *Achromobacter* | B | 8 | 16 | 0 | 0 |
| *Achromobacter* | C | 13 | 20 | 0 | 0 |
| *Achromobacter* | D | 18 | 11 | 0 | 0 |
| *Achromobacter* | E | 9 | 21 | 0 | 0 |
| *Ochrobactrum* | A | 15 | 29 | 0 | 0 |
| *Ochrobactrum* | B | 17 | 33 | 0 | 0 |
| *Ochrobactrum* | C | 17 | 58 | 0 | 0 |
| *Ochrobactrum* | D | 17 | 32 | 3 | 9.38% |
| *Ochrobactrum* | E | 18 | 43 | 0 | 0 |
| pKJK5::*gfp* transfer | | | | | |
| monoculture | A | 26 | 22 | 0 | 0 |
| monoculture | B | 58 | 18 | 0 | 0 |
| monoculture | C | 37 | 40 | 1 | 2.5% |
| monoculture | D | 24 | 16 | 0 | 0 |
| monoculture | E | 41 | 22 | 1 | 4.55% |
| *Pseudomonas* | A | 7 | 16 | 0 | 0 |
| *Pseudomonas* | B | 6 | 9 | 1 | 11.1% |
| *Pseudomonas* | C | 9 | 3 | 0 | 0 |
| *Pseudomonas* | D | 5 | 6 | 0 | 0 |
| *Pseudomonas* | E | 4 | 3 | 0 | 0 |
| *Stenotrophomonas* | A | 11 | 26 | 0 | 0 |
| *Stenotrophomonas* | B | 10 | 32 | 1 | 3.13% |
| *Stenotrophomonas* | C | 9 | 32 | 0 | 0 |
| *Stenotrophomonas* | D | 14 | 26 | 0 | 0 |
| *Stenotrophomonas* | E | 15 | 39 | 1 | 2.56% |
| *Achromobacter* | A | 7 | 14 | 0 | 0 |
| *Achromobacter* | B | 5 | 13 | 0 | 0 |
| *Achromobacter* | C | 6 | 16 | 0 | 0 |
| *Achromobacter* | D | 9 | 31 | 0 | 0 |
| *Achromobacter* | E | 11 | 28 | 0 | 0 |
| *Ochrobactrum* | A | 4 | 14 | 0 | 0 |
| *Ochrobactrum* | B | 3 | 19 | 0 | 0 |
| *Ochrobactrum* | C | 3 | 21 | 0 | 0 |
| *Ochrobactrum* | D | 6 | 20 | 0 | 0 |
| *Ochrobactrum* | E | 14 | 23 | 0 | 0 |

Table S2: Growth partner experimental values. P value refers to the probability of the treatment average being significantly different from the average of treatment 1.1 as assessed by the statistical model in **Figure 2A**.

| **Treatment (n=5)** | **Mean pKJK5::*gfp*^PL^-bearing**  ***Variovorax* fraction** | **Standard deviation** | **p value** |
| --- | --- | --- | --- |
| 1.1 V | 0.96 | 0.01 | NA |
| 2.1 PV | 0.71 | 0.06 | 1.2x10^-1^ |
| 2.2 SV | 0.53 | 0.06 | 1.1x10^-4^ |
| 2.3 AV | 0.93 | 0.06 | 1.0 |
| 2.4 OV | 0.87 | 0.04 | 1.0 |
| 3.1 PSV | 0.60 | 0.08 | 2.3x10^-3^ |
| 3.2 PAV | 0.77 | 0.07 | 7.7x10^-1^ |
| 3.3 POV | 0.71 | 0.16 | 1.1x10^-1^ |
| 3.4 SAV | 0.47 | 0.17 | 1.0x10^-6^ |
| 3.5 SOV | 0.64 | 0.11 | 9.2x10^-3^ |
| 3.6 AOV | 0.97 | 0.05 | 1.0 |
| 4.1 PSAV | 0.67 | 0.23 | 4.3x10^-2^ |
| 4.2 PSOV | 0.48 | 0.22 | 8.0x10^-7^ |
| 4.3 PAOV | 0.84 | 0.09 | 8.4x10^-1^ |
| 4.4 SAOV | 0.71 | 0.11 | 3.1x10^-2^ |
| 5.1 PSAOV | 0.83 | 0.19 | 6.6x10^-1^ |

Table S3: Growth partner experiment statistical model details. This table outlines the constituents of a statistical model fitted to the data of the experiment testing *Variovorax* pKJK5::*gfp*^PL^ maintenance in the presence of various growth partners. Significance values of individual model constituents were derived by Chi test. This model is a binomial model with logit link function and was further reduced by removal of non-significant (Pr>0.05) constituents for final data analysis for Figure 3.

Model function: V_fraction ~ Treatment + replicate + comp_P + comp_S + comp_A + comp_O

As only four (N-1) explanatory variables of species proportion could be fitted to the statistical model simultaneously (N=5), the probability of significance for comp_V was assessed after removal of the variable comp_O. This variable was chosen for removal as it did not reach significance with any combination of included explanatory variables.

| **Variable** | **Description** | **Probability of significance (Pr \|>Chi\|)** |
| --- | --- | --- |
| V_fraction | pKJK5::*gfp*^PL^-bearing fraction of *Variovorax* colonies | NA (response variable) |
| Treatment | Treatments from 1.1-5.1 as in Table S2 | 2.86 x 10^-11^ |
| replicate | Replicate 1-5 for each treatment | 0.79 |
| comp_P | Proportion of *Pseudomonas* within community. | 0.45 |
| comp_S | Proportion of *Stenotrophomonas* within community. | 0.0032 |
| comp_A | Proportion of *Achromobacter* within community. | 0.0016 |
| comp_O | Proportion of *Ochrobactrum* within community. | 0.20 |
| comp_V | Proportion of *Variovorax* within community. | 0.20 |

Table S4: Model estimate table. This table shows the model estimates of the statistical model fitted to data in Figure 2A. Model call: glm(formula = V_fraction ~ Treatment + comp_S + comp_A, family = binomial(link = "logit"), data = plot_df, weights = V).

Significance codes: 0 ‘***’ 0.001 ‘**’ 0.01 ‘*’ 0.05 ‘.’ 0.1 ‘ ’ 1

Residual standard error: 1.065 on 62 degrees of freedom

Pseudo R-squared: 0.8483776

F-statistic: 0.4635 on 17 and 62 DF, p-value: 0.9601

| Coefficients: | | | | | |
| --- | --- | --- | --- | --- | --- |
|  | Estimate | Std. Error | t value | Pr(>\|t\|) |  |
| (Intercept) | 3.13E+00 | 2.64E-01 | 11.853 | < 2e-16 | *** |
| Treatment2.1 | -2.25E+00 | 3.46E-01 | -6.5 | 1.57E-08 | *** |
| Treatment2.2 | -4.92E+00 | 7.33E-01 | -6.715 | 6.69E-09 | *** |
| Treatment2.3 | -3.61E+00 | 1.36E+00 | -2.665 | 0.009806 | ** |
| Treatment2.4 | -1.19E+00 | 3.38E-01 | -3.509 | 0.000842 | *** |
| Treatment3.1 | -4.04E+00 | 5.57E-01 | -7.256 | 7.74E-10 | *** |
| Treatment3.2 | -3.40E+00 | 7.04E-01 | -4.835 | 9.14E-06 | *** |
| Treatment3.3 | -2.27E+00 | 3.44E-01 | -6.586 | 1.11E-08 | *** |
| Treatment3.4 | -5.56E+00 | 7.56E-01 | -7.349 | 5.34E-10 | *** |
| Treatment3.5 | -3.97E+00 | 5.68E-01 | -6.996 | 2.18E-09 | *** |
| Treatment3.6 | -1.84E+00 | 8.21E-01 | -2.242 | 0.028537 | * |
| Treatment4.1 | -5.16E+00 | 9.76E-01 | -5.29 | 1.69E-06 | *** |
| Treatment4.2 | -4.24E+00 | 4.62E-01 | -9.18 | 3.66E-13 | *** |
| Treatment4.3 | -3.07E+00 | 6.33E-01 | -4.844 | 8.86E-06 | *** |
| Treatment4.4 | -4.45E+00 | 6.93E-01 | -6.425 | 2.10E-08 | *** |
| Treatment5.1 | -4.24E+00 | 9.21E-01 | -4.604 | 2.11E-05 | *** |
| comp_S | 2.45E+00 | 8.56E-01 | 2.861 | 0.005757 | ** |
| comp_A | 4.06E+00 | 1.49E+00 | 2.73 | 0.008225 | ** |

Figure S1: pKJK5 variants. Block arrows indicate ORFs, length not to scale. **a WT pKJK5**. Published by (Bahl *et al.*, 2007), genbank acc. AM261282.1. The indicated section is accessory gene load 2 spanning from nts 21682-33379. **b pKJK5::*gfp*^PL^** payload is inserted at position 22540 within *intI1* and consists of SpyCas9 and non-targeting sgRNA with constitutive promoters as well as GFPmut3b as in c. **c pKJK5::*gfp*** payload is inserted at position 23107 and consists of GFPmut3b with lacI-repressible promoter alongside kanamycin resistance gene *aphA*. Published by (Klümper *et al.*, 2015).


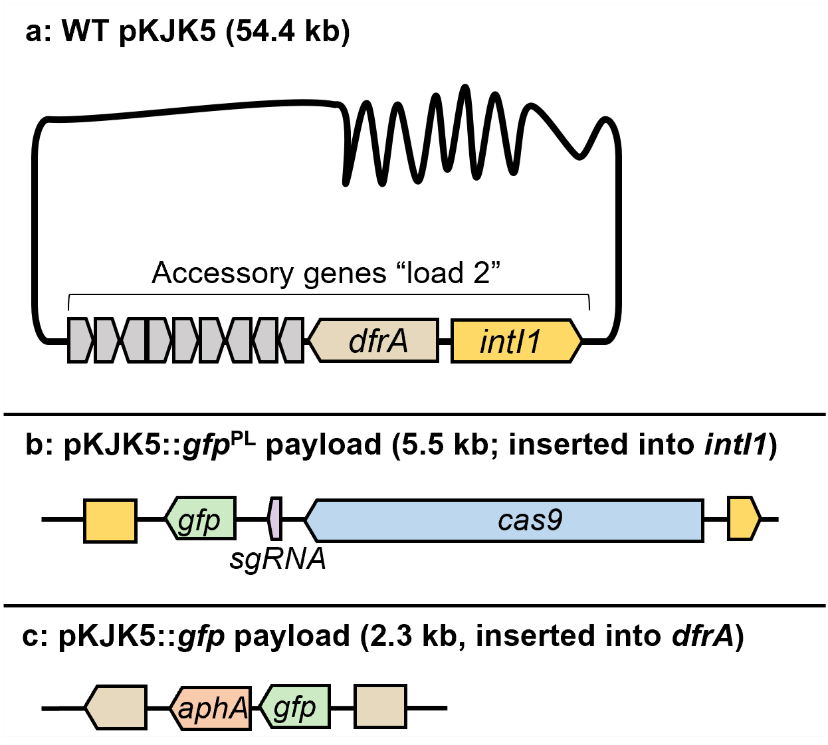


Figure S2: Design of Growth Partner Experiment. **A Strains at T0.** *Variovorax* is the only strain which carries pKJK5::*gfp*^PL^ or pKJK5::*gfp*. **B Various community treatments.** *Variovorax* was passaged in monoculture (1.1) or with 1-4 growth partners. **C Experimental setup.** T0 strains were co-incubated (in absence of selection) and transferred at T3. At T5, communities were plated onto KB agar and community composition was determined by counting colony morphologies. pKJK5-bearing *Variovorax* fraction was determined by analysing GFP expression on plates using a fluorescence lamp (not shown).


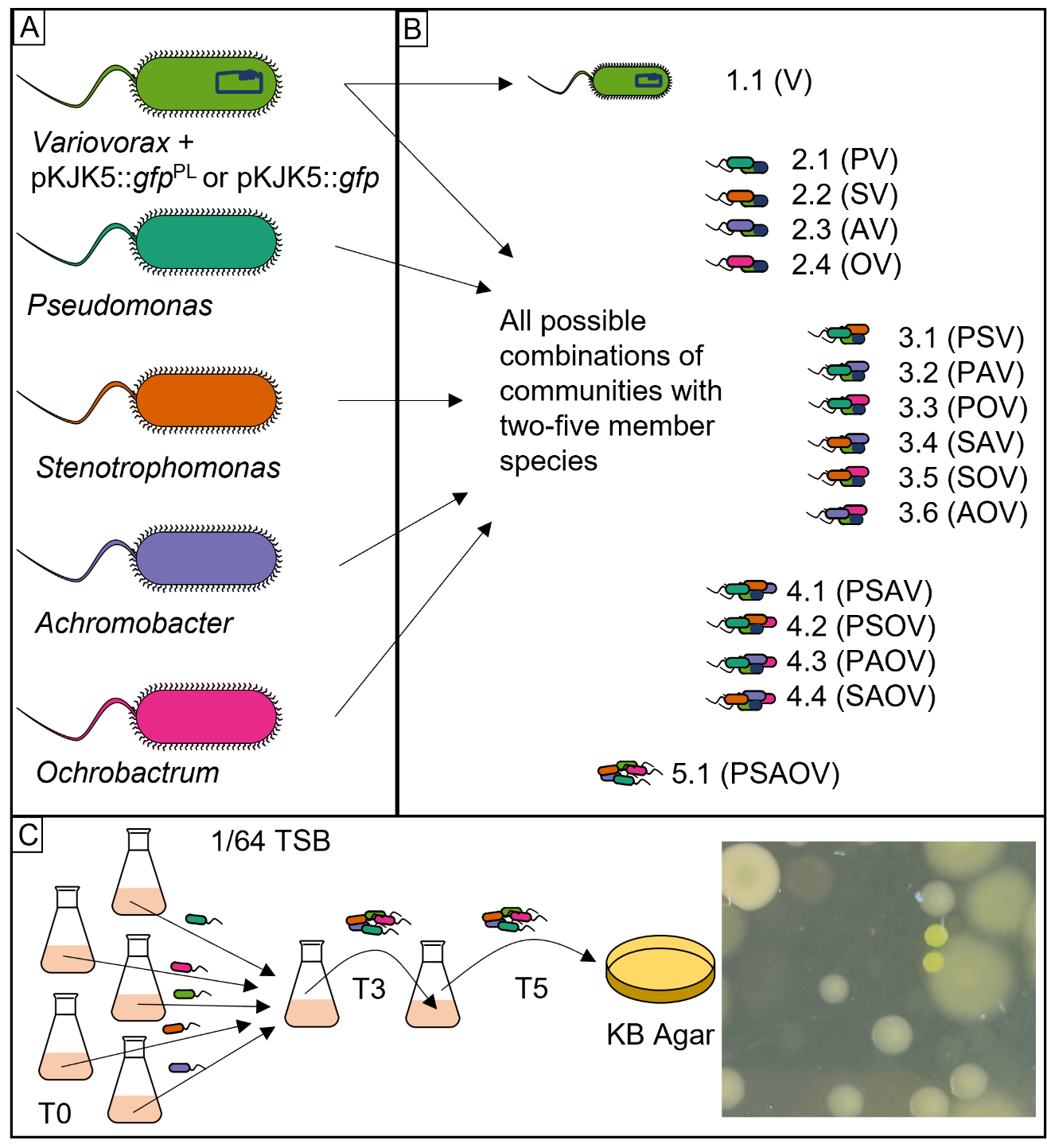


Figure S3: The relationship of community composition and *Variovorax* pKJK5::*gfp*^PL^ maintenance.

Community composition of all samples of various communities as in Figure 2A plotted against the GFP+ fraction of *Variovorax* colonies as a proxy of pKJK5::*gfp*^PL^-bearing Variovorax. Community composition is broken up into individual panels describing *Pseudomonas*, *Stenotrophomonas*, *Achromobacter*, *Ochrobactrum*, and *Variovorax* proportions of the whole community respectively. Counts of zero for each community constituent were removed for each panel.

Blue lines and shaded areas indicate fitted linear models with equations and R2 displayed in each panel. Linear model details are presented in **Table 1**. Of these five metrics, only *Stenotrophomonas* and *Achromobacter* proportion constitute significant terms of the statistical model fitted to the data; see **Table S2-4**.


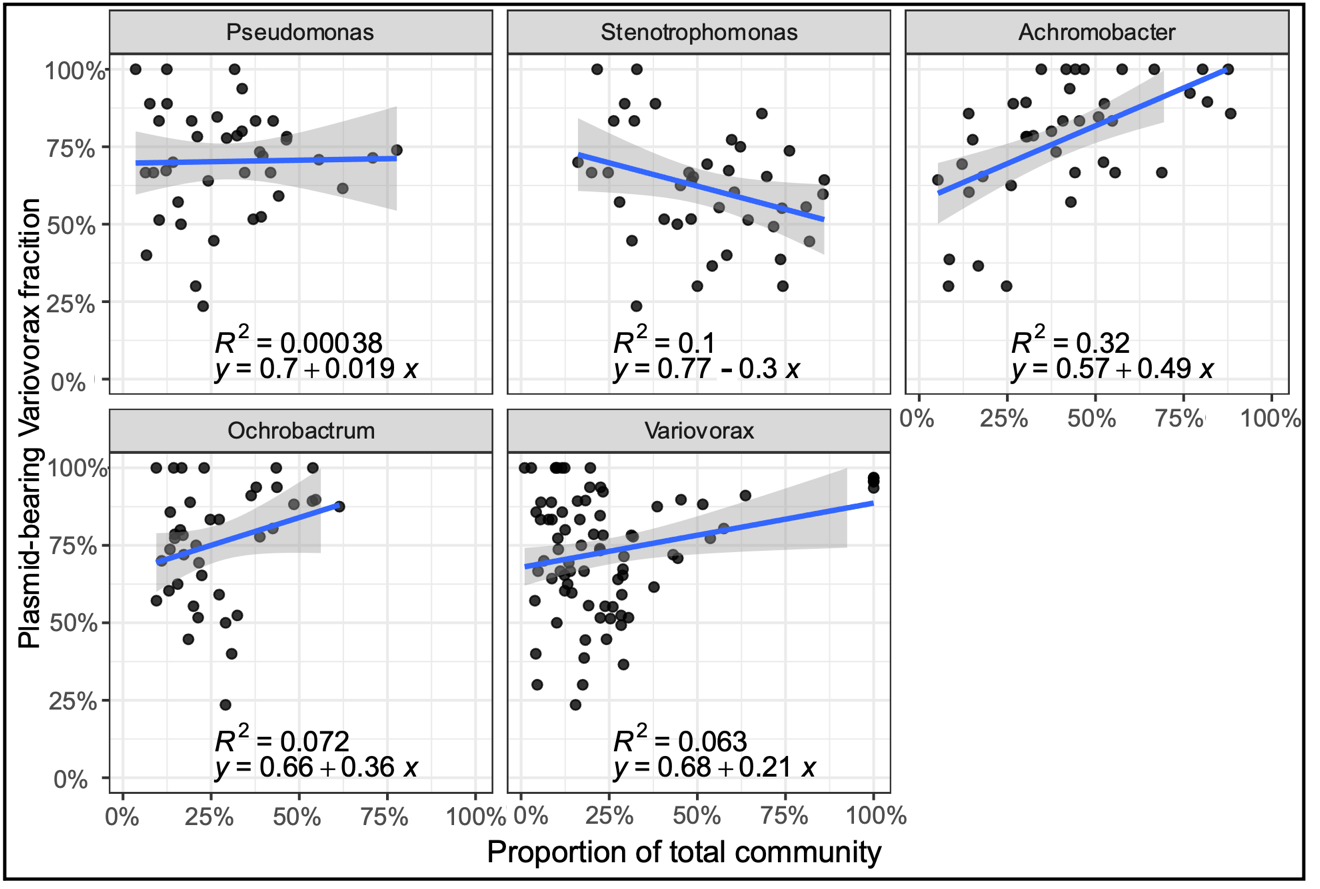


Figure S4: Pairwise correlations of community composition. Proportion of each species within each community where *Variovorax* carried pKJK5::*gfp*^PL^ plotted against each other; datapoints with community proportions containing 0 in each panel were removed (compare **Figure S3**). On the diagonal, a density line indicates the data point distribution of each species on an arbitrary scale. Above the diagonal, Pearson correlation coefficients are given. Significant pairwise correlations are indicated (*/**/***) and highlighted in boxes. *Variovorax* proportion was found to increase together with the presence of each growth partner; analogous to observed *Variovorax* density increases in pairwise competitions (Padfield *et al.*, 2020). *Achromobacter* proportion had a negative relationship with *Pseudomonas* and *Stenotrophomonas* proportion, which may in part explain the opposing effects these species have on *Variovorax* plasmid maintenance.

P – *Pseudomonas*; S – *Stenotrophomonas*; A – *Achromobacter*; O – *Ochrobactrum*; V – *Variovorax*.


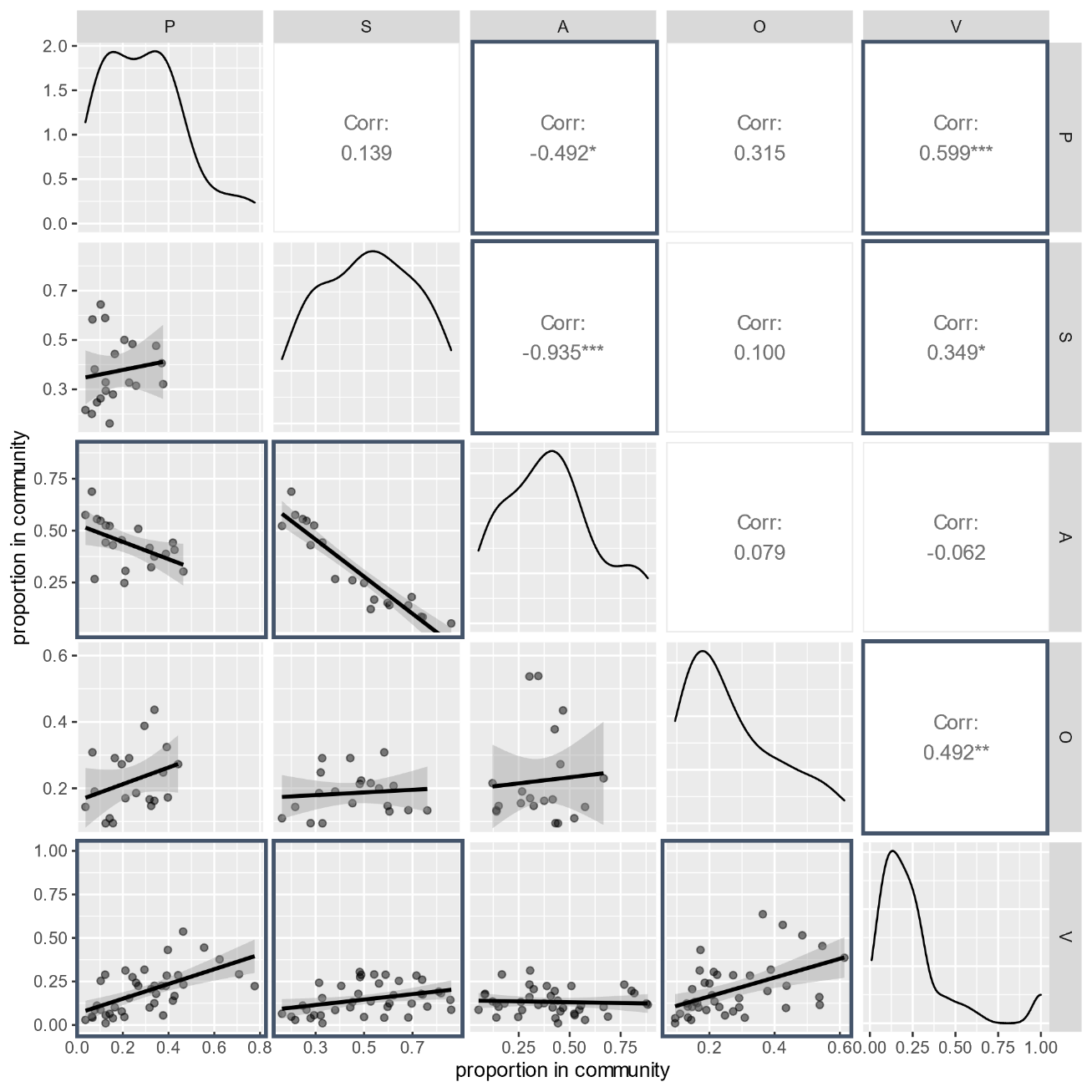


Figure S5: Species abundance after *Variovorax* competitions.

Absolute abundance of species after competition to assess pKJK5::*gfp*^PL^ (a; compare Figure 1A) or pKJK5::*gfp* (b; compare Figure 1B) fitness cost to *Variovorax*. Large circles and lines indicate mean ± standard deviation, small points indicate individual replicates of species abundance in CFU/mL after 5 days of co-culture. Abundance of both *Variovorax* chromosomal tag variants are displayed; Vg – *Variovorax* (Gm^R^) is the tag variant carrying pKJK5 in plasmid-bearing treatments; Vc – *Variovorax* (Cm^R^) is the tag variant remaining plasmid free in both treatments. Abundance of growth partners is shown where these are present. P – *Pseudomonas*; S – *Stenotrophomonas*; A – *Achromobacter*; O – *Ochrobactrum*.
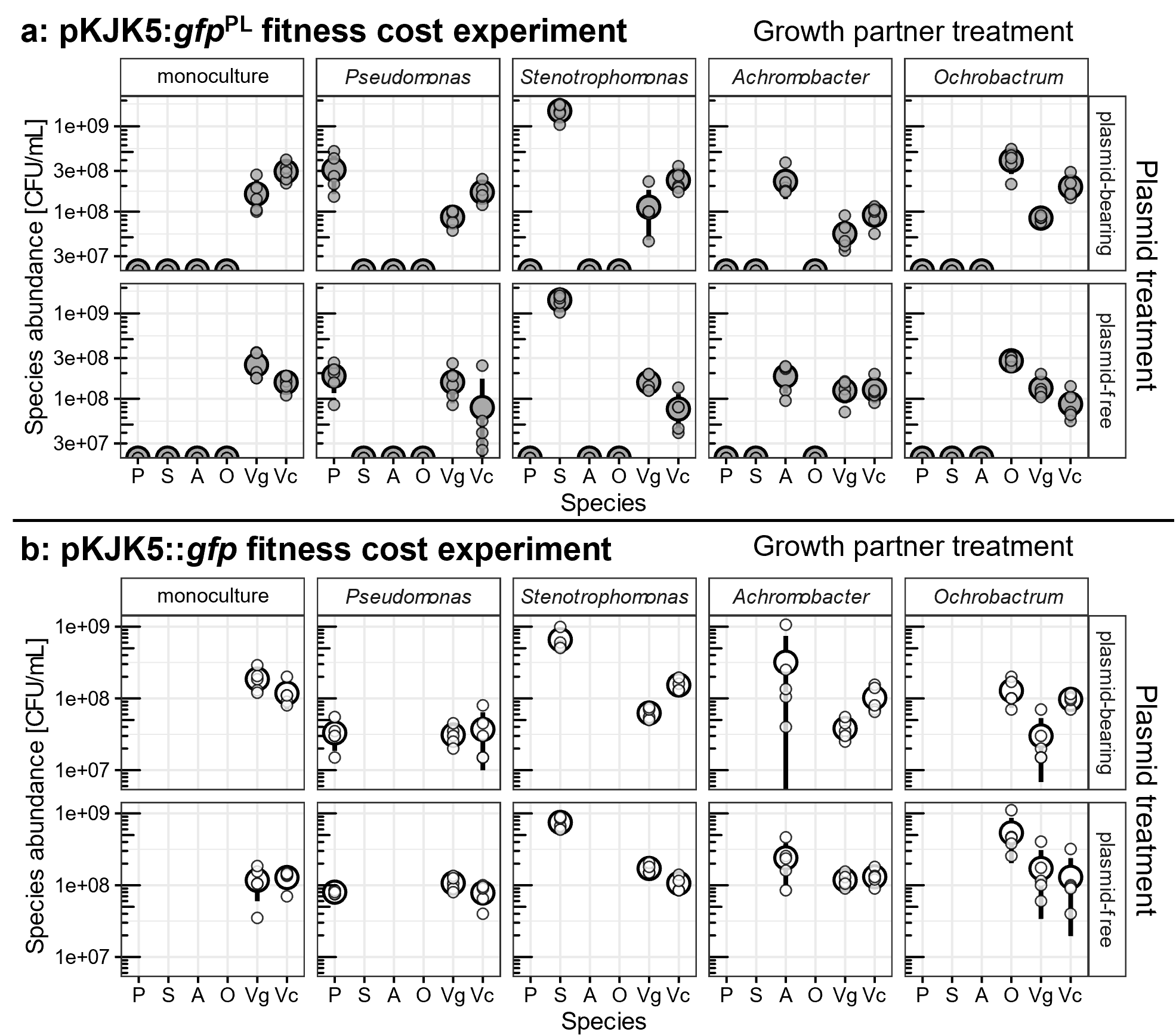


Figure S6 – Community composition of various communities.

Stacked bars show the proportion of each community member within its community. Transparent bars highlight community composition of each replicate a-e, opaque bars show the average community composition. P: *Pseudomonas*, S: *Stenotrophomonas*, A: *Achromobacter*, O: *Ochrobactrum*, V: *Variovorax.*

**a: various growth partner combinations as in Figure 2A**. Only *Variovorax* carries pKJK5::*gfp*^PL^. **b: various growth partner combinations as in Figure 2B.** Only *Variovorax* carries pKJK5::*gfp*. **c: full communities as in Figure 4.** All community members carry pKJK5::*gfp*^PL^/pKJK5::*gfp*.


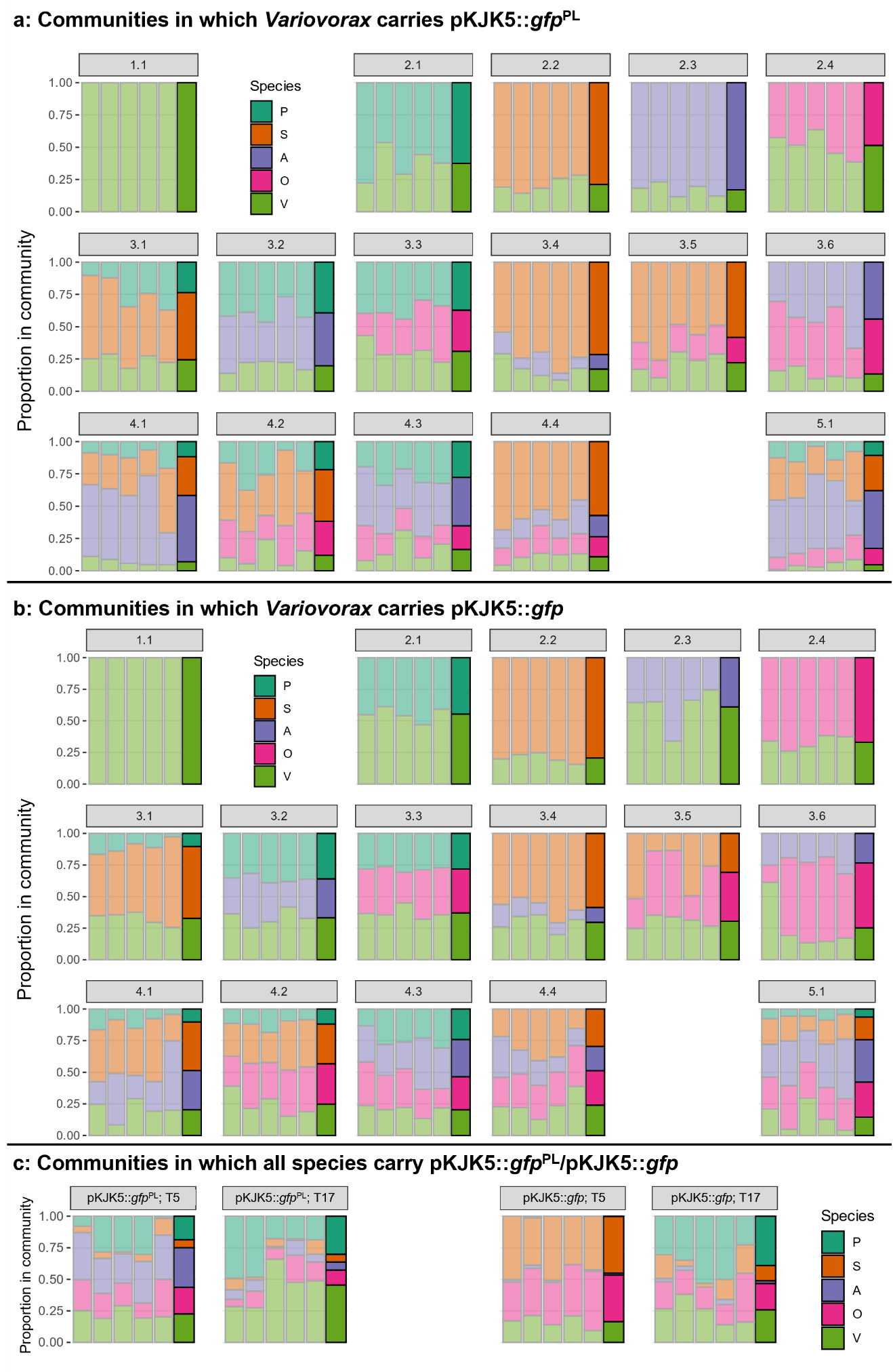


Figure S7 – Density of species in each community.

Total abundance of each species carrying the plasmid in monoculture and community context; matching the experimental data presented in Figure 4. Diamonds and lines show mean ± standard deviation of species abundance (CFU/mL) enumerated by colony morphology on non-selective plates. Individual replicates are shown as circles, N=2-5.


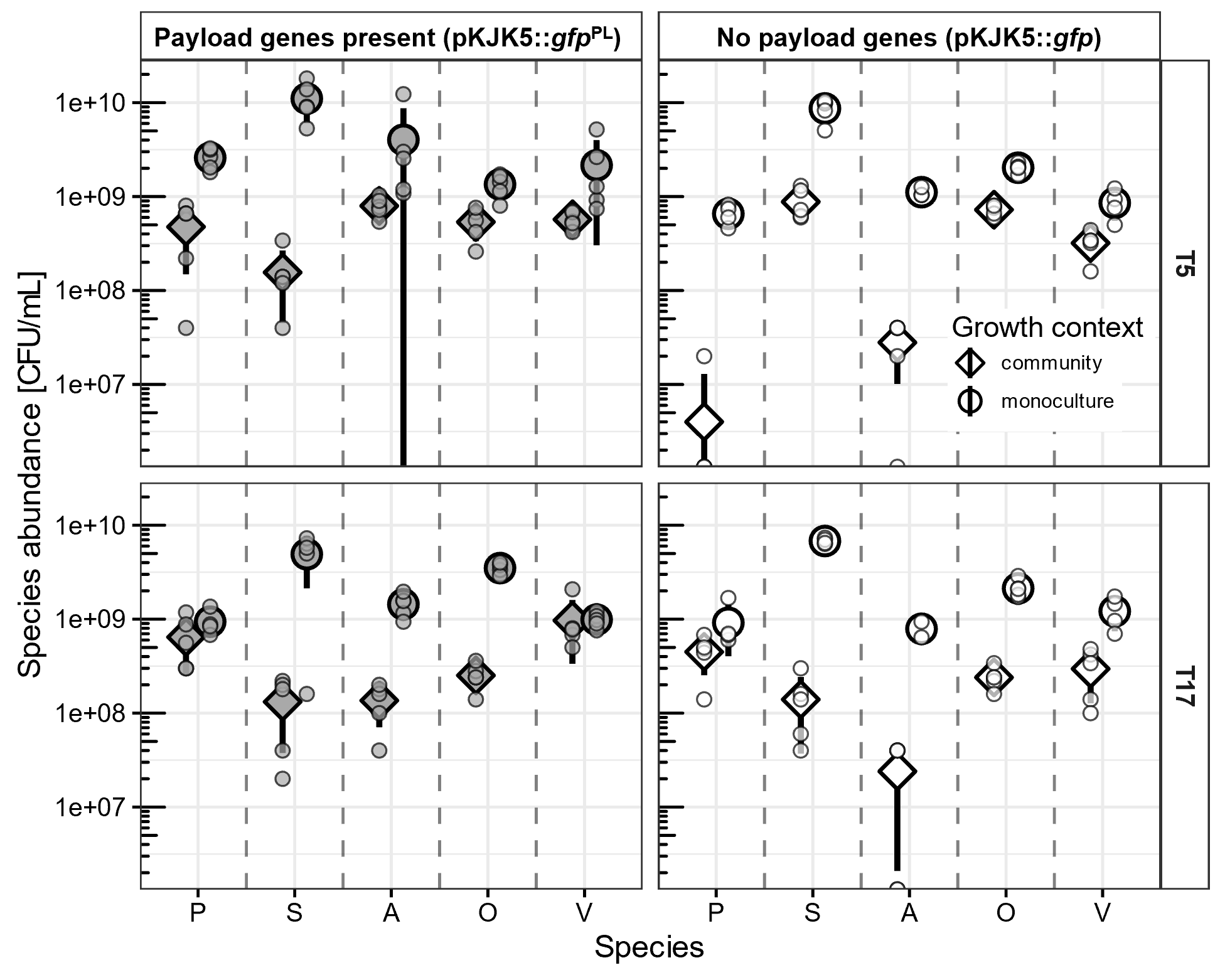


References:

Bahl, M.I., Hansen, L.H., Goesmann, A. and Sørensen, S.J. 2007. The multiple antibiotic resistance IncP-1 plasmid pKJK5 isolated from a soil environment is phylogenetically divergent from members of the previously established α, β and δ sub-groups. *Plasmid* 58: 31–43.

Klümper, U., Riber, L., Dechesne, A., Sannazzarro, A., Hansen, L.H., Sørensen, S.J. and Smets, B.F. 2015. Broad host range plasmids can invade an unexpectedly diverse fraction of a soil bacterial community. *The ISME Journal* 9: 934–945.

Padfield, D., Castledine, M., Pennycook, J., Hesse, E. and Buckling, A. 2020. Short-term relative invader growth rate predicts long-term equilibrium proportion in a stable, coexisting microbial community. *BioRxiv*.
